# A case-control study about markers of stress in normal-/overweight women with polycystic ovary syndrome and in controls

**DOI:** 10.3389/fendo.2023.1173422

**Published:** 2023-05-16

**Authors:** Marie-Louise Marschalek, Rodrig Marculescu, Christian Schneeberger, Julian Marschalek, Didier Dewailly, Johannes Ott

**Affiliations:** ^1^ Clinical Division of Gynecologic Endocrinology and Reproductive Medicine, Medical University of Vienna, Vienna, Austria; ^2^ Department of Laboratory Medicine and Institute of Immunology, Medical University Vienna, Vienna, Austria; ^3^ Faculté de Médecine, Université de Lille, Lille, France

**Keywords:** polycystic ovary syndrome, stress, saliva, biomarkers, cortisol, emotion, quality of life

## Abstract

**Background:**

Polycystic ovary syndrome (PCOS) is linked to an elevated risk of psychological disorders, decreased quality of life and emotional distress. Serum cortisol as a potential stress marker has been found to be increased in women with PCOS. The aim of this study was to evaluate both saliva stress markers and subjective psychological distress in women with PCOS.

**Methods:**

In a prospective case-control study, 31 PCOS women and 31 healthy controls were included. Salivary cortisol, and metanephrines were collected in the morning and in the evening. Emotional distress and quality of life were assessed by means of the Perceived Stress Scale (PSS-10) and the Short Form-36 (SF-36). Multivariable generalized linear models were applied to test the influence of various parameters on numerical outcome parameters.

**Results:**

After correction for age and body mass index (BMI), there were no statistically significant differences of salivary biomarkers between PCOS women and healthy controls (p>0.05). PCOS patients revealed significantly higher increased PSS total scores and lower quality of life in all SF-36 modules apart from pain (p< 0.05). The PSS total score was positively correlated to prolactin in PCOS women (r= 0.450; p= 0.011). In overweight/obese PCOS patients, a higher BMI, a higher Ferriman Gallwey score and higher age significantly predicted the PSS total score (p< 0.05).

**Conclusion:**

Stress measured by salivary biomarkers did not differ between PCOS women and healthy controls, whereas stress scores evaluated by questionnaires were significantly greater in women with PCOS. A higher BMI, hirsutism and a higher age seem to be the main modulators of subjective stress in PCOS. Prolactin might serve as a biomarker for chronic stress in PCOS women.

## Introduction

Polycystic ovary syndrome (PCOS) is the most widespread female endocrinopathy, affecting 5-10% of women in the reproductive age. It is primarily characterized by ovulatory dysfunction and androgen excess. However, women with PCOS also fall prey to psychological distress, due to body dissatisfaction and impaired health-related quality of life. This psychological distress may manifest itself as depression and anxiety (1, 2) imposing a substantial health care burden. A higher prevalence of anxiety and depressive disorders in women with PCOS has been confirmed (3). The extents of these diseases could be connected to higher stress levels, which is also widely encountered in women with PCOS (4, 5). The stress response is modulated by the hypothalamus-pituitary-adrenal (HPA) axis and the sympathetic nervous system by synthesizing and releasing adrenal glucocorticoids and catecholamines. A recent meta-analysis of 41 studies reports cortisol to be elevated in PCOS subjects (6). However, the overall effect is accounted for a few large studies with the majority of included studies not reporting significant differences. Further, there are variations in the methodological characteristics, in particular of cortisol sampling, with most studies evaluating cortisol in the serum and only a few attempting to assess cortisol in saliva. Notably, the assessment of salivary biomarkers has received recognition because of the relaxed and non-invasive self-collection, as well as the easy handling of the saliva samples (7–9). Salivary cortisol represents an established tool for evaluating acute and chronic stress responses, as increased secretions of salivary stress biomarkers under different distressing conditions have been demonstrated (10–12). However, free metanephrines reflect the sympathetic/adrenomedullary system of the stress response.

The international evidence-based guidelines for the assessment and management of PCOS emphasize the importance of considering quality of life and stress in PCOS research, and the application of the correct methods in clinical care in order to recognize and highlight the priorities of patients (13). Appropriate evaluation, identification of high-risk individuals and management of stress at an early stage may prevent the onset of secondary disorders like depression and anxiety, and thereby facilitate positive, long-term mental health outcome and have a favorable impact on the health and financial burden on the health-care system (14, 15). To our knowledge, no studies have yet examined the association of perceived stress and cortisol secretion in PCOS women.

Moreover, sympathetic activity is increased in subjects with obesity (16) and there seems to be a complex and bidirectional relationship between sympathetic activity and insulin resistance (17). Given that insulin resistance is common in PCOS with a prevalence of 75-95% according to clamp studies (18), that the PCOS prevalence increases with obesity (19) and that the risk for insulin resistance and type 2 diabetes is higher in overweight and obese PCOS women (20), one could hypothesize that sympathetic activity and perceived stress might differ between lean/normal weight and overweight/obese PCOS women. It has already been reported that concerns about weight were associated with a decreased quality of life in patients with PCOS (21).

Thus, in light of the prevalence of distress among PCOS patients, our aim was to evaluate both self-perceived severity of symptoms measured by the Perceived Stress Score (22) and Short Form-36 (23), and the salivary biomarkers cortisol and free metanephrines. As a secondary study aim, we also focused on differences between lean/normal weight and overweight/obese PCOs patients.

## Materials and methods

### Study population and setting/study design

This was a prospective case-control study at the Clinical Department of Gynecologic Endocrinology and Reproductive Medicine of the Medical University of Vienna, Austria. The study was approved by Institutional Review Board of the Medical University of Vienna (IRB number 1804/2016), and each subject provided written informed consent to participate. Thirty-one non-infertile patients diagnosed with PCOS and 31 healthy female controls were recruited. Patients with PCOS were subsequently divided into an overweight/obesity (BMI > 25) group and a lean/normal-weight (BMI ≤ 25) group. Control subjects were women with a regular menses and without any signs of clinical or biochemical hyperandrogenism, who did not use any hormonal contraception. Diagnosis of PCOS was made according to the revised Rotterdam criteria (24), having two of the following criteria: 1) biochemical and/or clinical hyperandrogenism; 2) polycystic ovaries seen on transvaginal ultrasound; 3) an- or oligoovulation (24). Furthermore, participants were eligible if they met each of the following criteria: age 18-40 years; able to read, understand and sign the informed consent; not having non-classic adrenal hyperplasia; not having PCOS-specific treatment within the last three months before study enrollment.

Study participants were recruited from the outpatient clinic of the Department of Gynecologic Endocrinology and Reproductive Medicine of the Medical University of Vienna. All patients were referred by a gynecologist, seen by a specialist at our clinic and underwent medical routine examination including laboratory measurements. After initial diagnosis of PCOS, the patients were recruited and, if they were willing to participate, they were invited for an extra study visit.

The recruitment of participants was initiated in January 2017. During the extra study-visit, participants were given two questionnaires, which were completed by themselves on-site. Serum samples of participants with PCOS were collected beforehand as part of clinical routine. All participants were instructed to collect saliva samples. Furthermore, clinical parameters were evaluated and documented in the case report form.

### Measures

Blood samples were analyzed for total and free testosterone, the free androgen index (FAI) calculated as 100 x (total testosterone/sex hormone binding globulin), androstenedione, dehydroepiandrosterone sulfate (DHEAS), the ratio of luteinizing hormone (LH)/follicle-stimulating hormone (FSH), antimullerian hormone (AMH), sex hormone binding globuline (SHBG), prolactin and cortisol-binding globulin (CBG). Insulin resistance was defined by a HOMA-IR >2.5, calculated as fasting insulin (mU/l) * fasting glucose (mg/dl)/405 (25).

Saliva samples of salivary cortisol and free metanephrines were collected with the Salivette sampling device (Sarstedt, Germany) in the morning, within 30 minutes after awakening, and in the evening, by the participants themselves. Participants returned samples within a week. Samples were then centrifuged and stored at -80°C until assayed.

Perceived stress was assessed with the German version of the 10-item Perceived Stress Scale (PSS-10) which calculates the point at which events are considered incontrollable, unpredictable and/or overloading (22). The German version of the PSS-10 has also been validated (26, 27). Answers to all questions were rated on a five-point Likert scale (0= never, 4= very often). The scale correlates with different psychological measures specifically depression, anxiety as well as decreased satisfaction with self, job and life in general. A score of “perceived helplessness subscale” and a score of “perceived self-efficacy subscale” is obtained. The total Perceived Stress Scale (PSS-10) score is the sum of all “perceived helplessness subscale” items and reversed “perceived self-efficacy subscale” items. Quality of life was assessed with the German version of the Short Form-36 (SF-36), an established and validated 36-item instrument including a total of eight subscales (Physical Function, Physical Role Function, Emotional Role Function, Energy/Fatigue, Emotional wellbeing, Social Function, Bodily Pain, General Health (28). According to the guidelines, scores were converted to a 0-100 scale. Higher values of the transformed scale indicate better health status. Lower values indicate lower health status, show lower functional limitation, distress and further social and role disability.

In addition, we assessed clinical information of PCOS. Weight and height were calculated by weight/height squared (kg/m2) in all patients to BMI. The clinical assessment of hirsutism was determined using the Ferriman-Gallwey scoring system (29). Acne vulgaris was evaluated by use of the global acne grading system (30).

Blood samples were obtained during the early follicular phase visit (cycle days 2-5). All biochemical analyses were carried out at the Department of Laboratory Medicine, Medical University of Vienna using CE IVD labeled assays according to ISO 15189 standards. As reported previously (31), prolactin, DHEAS and cortisol measurements were performed with the corresponding Cobas electrochemiluminescence immunoassays (ECLIA) on Cobas e 801 immunologyanalyzers (Roche Diagnostics, Mannheim, Germany). Metanephrine and Normetanephrine were measured by ELISAs (LDN, Nornhorn, Germany) and CBG by RIA (DiaSource, Louvain-la-Neuve, Belgium).

Polycystic ovarian morphology was defined by a number of follicles per ovary >12. For vaginal ultrasound, an “Aloka Prosound 6” ultrasound machine and an “UST-9124 Intra Cavity transducer” (frequency range 3.0 - 7.5 MHz; Wiener Neudorf, Austria) were used (31).

### Statistical analysis and sample size calculation

The sample size was calculated to detect a 0.2 difference in saliva cortisol levels between PCOS women and controls with an expected SD of 0.25 at a power of 80% and an alpha of 0.05. This required 26 participants in each group. Due to the medium effort that was expected of the participants, we anticipated a dropout rate of 20%, five participants respectively per group. To account for dropouts, we therefore aimed to recruit 31 participants per group. Accordingly, the total sample size was estimated with 62.

### Statistical analysis

Data are presented as median and interquartile range for numerical parameters and as number and frequency for categorical parameters. The SF-36 and PSS-10 questionnaires were scored and analyzed according to the published guidelines. To test differences between groups, numerical parameters were compared using the unpaired t-test (in case of a normal distribution) or the Welch-test. Pearson correlation was used to examine relationships between numerical parameters. Statistical analyses were performed in SPSS 24.0 (IBM, Vienna, Austria). For comparison of groups with correction for age and BMI, univariable binary logistic regression models were used, where age and BMI were also entered as covariates. Multivariable generalized linear models were applied to test the influence of various parameters on numerical outcome parameters. For these analyses, ß-values (B) with their standard deviations (standard errors) as well as 96% confidence intervals (95% CI) and the Wald’s tests are provided. A p-value < 0.05 was considered statistically significant.

## Results

**Table 1** provides details about basic patient and clinical characteristics in PCOS patients and healthy controls. A higher BMI was found for women with PCOS. Moreover, PCOS patients revealed a higher median Ferriman Gallwey score, higher levels of LH, testosterone, DHEA-S, prolactin, AMH, an increased FAI, and higher rates of polycystic ovarian morphology on ultrasound (*p<* 0.05).

**Table 1:** Basic patient and clinical characteristics of PCOS women and healthy controls.

	PCOS patients	Controls	p
Age (years)	25 (22,30)	28 (24,31)	0.071
BMI (kg/m^2^)	24.9 (21.5;35.5)	21.5 (19.5;23.9)	0.001
Insulin resistance (HOMA-IR >2.5)	20 (64.5)	–	–
Ferriman Gallwey Score	10 (5,15)	0 (0;0)	<0.001
GAGS	4 (4;11)	0 (0;6)	0.198
LH (mlU/mL)	12.6 (8.5;14.6)	5.2 (3.4;7.1)	<0.001
FSH (mlU/mL)	5.6 (4.9;7.5)	5.9 (4.7;8.1)	0.548
LH: FSH ratio	2.18 (1.51;3.09)	0.93 (0.55;1.4)	<0.001
Testosterone (ng/mL)	0.51 (0.39;0.72)	0.25 (0.16;0.31)	<0.001
SHBG (nmol/L)	35.3 (29.1;73.7)	82.3 (68;114.6)	0.001
Free androgen index	1.15 (0.72;2.53)	0.29 (0.20;0.38)	<0.001
DHEA-S (µg/ml)	3.17 (2.53;4.33)	2.19 (1.81;2.80)	0.001
Prolactin (ng/mL)	13.1 (8.9;17.8)	9.5 (8.3; 13.1)	0.017
AMH (ng/mL)	7.83 (5.94;11.00)	3.01 (2.03-4.07)	<0.001
Presence of polycystic ovarian morphology on ultrasound	25 (80.6)	4 (12.9)	<0.001

Data are provided as median (interquartile range) for numerical parameters and as number (frequency) for categorical parameters

–, not applicable.

BMI, body mass index; HOMA-IR, HOMA index of insulin resistance; GAGS, global acne grading system; LH, luteinizing hormone; FSH, follicle stimulating hormone; DHEA-S, dehydroepiandrosterone-sulfate; SHBG, sexual hormone binding globulin; AMH, anti-Mullerian hormone.

When PCOS women were compared to controls after correction for age and BMI (**Table 2**), there were no differences between the two groups concerning laboratory stress markers. In contrast, women with PCOS revealed significantly increased PSS total scores as well as significantly lower quality of life in all SF-36 modules apart from pain.

**Table 2:** Salivary and serum stress markers as well as main results of the SF-36 and PSS questionnaires in PCOS women and controls.

	PCOS patients	Controls	p*
Morning saliva cortisol (µg/dL)	0.326 (0.201;0.490)	0.416 (0.217;0.529)	0.798
Evening saliva cortisol (µg/dL)	0.054 (0.054;0.054)	0.054 (0.054;0.054)	0.168
Morning saliva metanephrines (pg/mL)	13.7 (10.0;28.2)	11.2 (8.2;27.6)	0.937
Evening saliva metanephrines (pg/mL)	12.5 (7.1;17.5)	14.2 (8.8;19.7)	0.338
Morning saliva normetanephrines (pg/mL)	175.0 (58.6;370.5)	187.0 (86.2;340.8)	0.182
Evening saliva normetanephrines (pg/mL)	118.0 (80.9;336.0)	136.0 (93.9;256.5)	0.466
Serum CBG (µg/mL)	51.0 (47.1;56.8)	54.0 (51.6;59.4)	0.214
PSS: PHS score	18 (15;22)	14 (11;16)	<0.001
PSS: PSE score	11 (10;13)	15 (13;16)	<0.001
PSS: total score	7 (4;11)	0 (6;4)	<0.001
SF-36: Physical functioning	90 (80-100)	100 (95-100)	0.002
SF-36: Role functioning/physical	100 (75-100)	100 (100-100)	0.025
SF-36: Role functioning/emotional	66.6 (33.3-100)	100 (67-100)	0.007
SF-36: Energy/fatigue	40 (35-50)	55 (50-65)	<0.001
SF-36: Emotional wellbeing	64 (48-72)	76 (60-80)	<0.001
SF-36: Social functioning	75 (62.5-87.5)	87.5 (50-100)	0.021
SF-36: Pain	77.5 (67.5-100)	90 (68-100)	0.698
SF-36: General health	60 (50-75)	80 (70-90)	0.002

Data are provided as median (interquartile range).

*p was adjusted for age and BMI.

PSS, Perceived Stress Scale; PHS, perceived helplessness subscale; PSE, perceived self-efficacy subscale; SF-36, Short Form-36.

**Figure 1:**
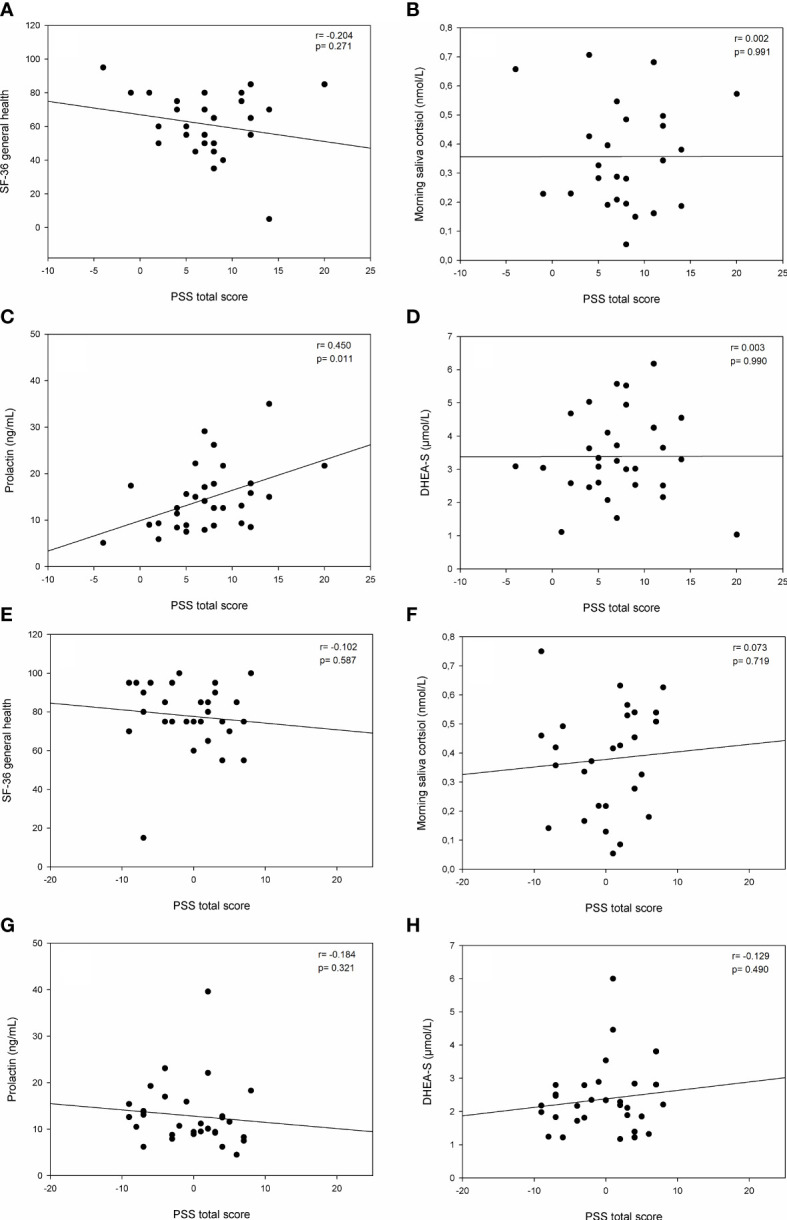
Correlation analyses between the Perceived Stress Scale’s total score and general health/stress-specific parameters. **(A)** PSS total score versus SF-36 general health in PCOS patients; **(B)** PSS total score versus morning saliva cortisol (nmol/L) in PCOS patients; **(C)** PSS total score versus Prolactin (ng/mL) in PCOS patients; **(D)** PSS total score versus DHEA-S (µmol/L) in PCOS patients; **(E)** PSS total score versus SF-36 general health in controls; **(F)** PSS total score versus morning saliva cortisol (nmol/L) in controls; **(G)** PSS total score versus Prolactin (ng/mL) in controls; **(H)** PSS total score versus DHEA-S (µmol/L) in controls.

Correlation analyses were performed between the PSS total score and SF-36 general health as well as possible salivary and serum stress markers, namely morning saliva cortisol, DHEAS and prolactin in PCOS patients (**Figures 1A–D**) and controls (**Figures 1E–H**) separately. The only significant correlation was found for the PSS total score and prolactin in PCOS women (*r=* 0.450; *p=* 0.011; **Figure 1C**). Notably, there was no significant correlation between SF-36 general health and the PSS total score, neither in PCOS patients (**Figure 1A**) nor in controls (**Figure 1E**).

In a next step, we evaluated whether PCOS-typical parameters were associated with two main outcome parameters, namely the PSS total score and the overall quality of life in SF-36, by the use of generalized linear models. Age, BMI, the Ferriman Gallwey Score (for PCOS patients), total testosterone and AMH were included into these models. When focusing on the PSS total score (**Table 3**), only a higher BMI was linked to increased stress in controls (*n=* 31), whereas none of the parameters was found to be of significant influence in the total PCOS population (*n=* 31). Similar results were found in lean/normal weight PCOS women (*n=* 15) despite a non-significant trend for testosterone and higher perceived stress. However, in overweight/obese PCOS patients a higher BMI, a higher Ferriman Gallwey score and higher age were significantly associated with stress (*p<* 0.05).

**Table 3:** Generalized linear models for the Perceived Stress Scale’s total score.

	Controls *(n= 31)*			PCOS: all patients *(n= 31)*			PCOS: lean and normal weight *(n= 15)*			PCOS: overweight and obese *(n= 16)*		
	*ß*	*SD (ß)*	*p*	*ß*	*SD (ß)*	*p*	*ß*	*SD (ß)*	*p*	*ß*	*SD (ß)*	*p*
Constant	-0.035	5.845	0.995	-3.112	5.316	0.558	0.596	10.685	0.956	-25.119	4.686	<0.001
Age (years)	0.301	0.192	0.118	0.176	0.170	0.300	0.256	0.189	0.175	0.351	0.150	0.019
BMI (kg/m2)	-0.533	0.167	0.001	0.145	0.107	0.174	0.084	0.405	0.835	0.532	0.087	<0.001
Ferriman Gallwey Score	–	–	–	0.076	0.106	0.473	-0.068	0.104	0.509	0.334	0.098	<0.001
Testosterone (ng/mL)	8.696	6.877	0.206	-4.982	3.206	0.120	8.407	4.392	0.056	0.354	2.412	0.883
AMH (ng/mL)	0.271	0.604	0.653	0.424	0.223	0.057	0.406	0.223	0.069	-0.206	0.251	0.410

–, not applicable.

Concerning overall quality of life in the SF-36 questionnaire (**Table 4**), a similar pattern was found. This time, an increased BMI was the major modulator associated with a decreased quality of life and this was the case in the whole PCOS group as well as in overweight/obese PCOS patients (*p<* 0.05).

**Table 4:** Generalized linear models for total health in SF-36.

	Controls *(n= 31)*			PCOS: all patients *(n= 31)*			PCOS: lean and normal weight *(n= 15)*			PCOS: overweight and obese *(n= 16)*		
	*ß*	*SD (ß)*	*p*	*ß*	*SD (ß)*	*p*	*ß*	*SD (ß)*	*p*	*ß*	*SD (ß)*	*p*
Constant	598.665	100.519	<0.001	512.710	100.092	<0.001	498.977	198.176	0.012	741.939	137.744	<0.001
Age (years)	-3.860	3.177	0.224	-1.254	3.085	0.684	-3.293	3.507	0.348	2.332	4.421	0.598
BMI (kg/m2)	0.027	1.859	0.988	-5.754	1.919	0.003	-4.629	7.509	0.538	-13.897	2.554	<0.001
Ferriman Gallwey Score	–	–	–	-1.796	1.914	0.348	-2.255	1.923	0.241	-1.655	2.891	0.567
Testosterone (ng/mL)	-230.095	121.519	0.058	53.526	57.806	0.354	-2.369	81.461	0.977	99.349	70.893	0.161
AMH (ng/mL)	-14.834	10.203	0.146	-2.167	4.087	0.596	2.952	4.136	0.475	-7.494	7.367	0.309

–, not applicable.

## Discussion

The PCOS patients in our study population revealed typical general and PCOS-specific characteristics with a median age of 25 years, a rate of overweight/obesity of about 52%, and increased testosterone, DHEA-S and LH levels (**Table 1**). Notably, controls were significantly older and had a lower BMI. Thus, all results of the laboratory stress markers and the questionnaires had to be corrected for age and BMI when compared between PCOS women and controls (**Table 2**). We did not find differences in laboratory stress biomarkers between PCOS women and controls. Despite the fact that salivary stress markers have been implemented to assess distress in several studies (7, 12, 32, 33), with salivary cortisol being the most established biological marker in stress research, only few authors have attempted to evaluate salivary stress markers in PCOS women so far (34–36). Tock et al. similarly found no hyperactivity of the HPA axis in PCOS women compared to controls. However, non-obese PCOS women had higher salivary cortisol levels when compared to obese PCOS women in the mentioned study (36). On the other hand, an overactivity of the HPA-axis triggered by stressful stimuli was seen as a characteristic of hirsute adolescents, detected by increased salivary glucocorticoid measurements (35).

Noteworthy, studies about cortisol production in PCOS have presented heterogeneous results. While some studies found elevated serum cortisol levels (37–39), others found normal levels (40–42). A recent meta-analysis performed by Benjamin et al. summarized that women with PCOS had higher cortisol levels than controls. However, significant heterogeneity existed across the various studies included. Moreover, the overall effect was accounted for a few large studies, whereas the majority of included studies did not report significant differences (6).

As stated previously, in addition to the objective biochemical assessment of stress, we equally performed subjective evaluation of mental and general health and self-perceived severity of symptoms. Women with PCOS had significantly higher total stress scores, higher perceived helplessness scores and lower perceived self-efficacy scores in the PSS compared to healthy controls. This is in line with other studies, such as Khafagy et al., who found a significant difference in PSS scores among adolescents with and without PCOS (43). Furthermore, significant differences of health related quality of life in terms of physical health and emotional health using the SF-36 were found on the majority of subscales in our study, showing lower scores among PCOS women and therefore indicating a compromised quality of life. Similarly, a recent case-control-study observed significant differences in various domains of the short form health survey-36 between PCOS and healthy control cases (44). Comparable results have been previously published and our findings confirm the overall convergence that women with PCOS are at increased risk of emotional distress and diminished quality of life (5, 45–49). A recent systematic review and meta-analysis conducted by Yin et al. concluded that women with PCOS experience a lower quality of life and more frequently suffered from depression and anxiety (49), which supports the well-known and well-established effect of PCOS on general health and quality of life (48).

Despite the fact that sample sizes were small for generalized linear models, a higher BMI and hirsutism seemed to influence perceived stress mainly in obese PCOS women (**Table 3**). However, with a focus on the comparably small ß-values in the lean/normal weight PCOS group, BMI and the Ferriman Gallwey Score did not seem to be of major influence on the PSS total score and overall quality of life. Although being overweight is known to be a contributor to an impaired quality of life and depression in the general public, there are conflicting results concerning the influence of BMI on mental distress in PCOS women. Previous studies reported perceived stress scores to be independent of BMI (43) and that an obesity category was not connected to emotional quality of life (5). Other studies demonstrated that an increased BMI did alter health related quality of life (44). The study by Karsten et al. looked at whether obese women suffered specifically if PCOS was detected, with a particular mental health impact. However, despite the fact that these issues are related to the PCOS condition, the authors concluded that the impaired mental quality of life, anxiety, depression and physical quality of life seemed to be more connected to the obesity rather than to the PCOS condition (50). Moreover, females with PCOS report lower body image satisfaction compared to the females without PCOS (51, 52). This may be due to being overweight, or to the androgen related disorders such as hirsutism affecting women’s feelings of attractiveness (53). Our findings demonstrate a possible influence of an increased BMI and hirsutism on mental health in particular of obese PCOS women, which could be due to the impact on femininity and body image.

Interestingly, reproductive and metabolic PCOS characteristics are associated with specific PCOS susceptibility loci. As reported, increasing BMI appeared to be causal for PCOS development, whereas having PCOS did not affect BMI (54). While these data support a genetic background, PCOS development also seems to be induced or at least intensified by overweight/obesity. Thus, one could assume different pathophysiologic pathways in overweight/obese PCOS women, more based on a (genetically) higher BMI, and in lean/normal weight PCOS patients, more likely due to a direct genetic risk for PCOS. Likewise, the source of stress could differ between these populations. Notably, a shared genetic basis of PCOS with psychiatric diseases has been refuted (55). However, our data suggest that the higher BMI itself and hirsutism were of major influence on stress in overweight/obese women, whereas in lean/normal weight PCOS patients testosterone as a marker of overall disease severity showed a trend (*p=* 0.056; **Table 3**).

In addition to these findings, prolactin was the only serologic stress marker, which was correlated to the PSS total score. In detail, there was a positive correlation. Prolactin has been mentioned as a serum parameter possibly elevated in patients with chronic stress like burn out (56). This would also explain the significantly higher prolactin levels in PCOS patients (*p=* 0.017; **Table 1**). From a pathophysiologic point of view, chronic stress induces an intense cortisol production. For the production of cortisol, the POMC-neurons must produce ACTH. By doing this, they also secrete GABA and glutamate. GABA acts a stimulator of prolactin and can therefore lead to an increased prolactin secretion (57). We are aware of the fact that cortisol levels did not differ between PCOS patients and controls, a fact, which we find hard to comment on. In addition to prolactin, PCOS women revealed higher DHEA-S levels, which have also been claimed to be linked to chronic stress (56), but do not seem to be specific for a stress response rather than for the hyperandrogenemic state.

To our best knowledge, this study is the first trial evaluating both salivary stress biomarkers including salivary cortisol measurement and self-perceived severity of symptoms of stress and quality of life in adult PCOS women in one population. Further strengths of our study include the use of validated general and condition- specific questionnaires and an excellent participant retention. Although adequately powered, we are aware of the fact that the study sample size was nevertheless small, and that future studies with a larger sample size are required. A further limitation is that healthy controls had a slightly, but significantly, lower BMI than the PCOS participants at baseline, despite a proactive matching strategy. We realize that the generalizability of our trial results is limited by the homogenous population. Moreover, although salivary measures have proven to be a reliable method, it is accepted that they only provide information pertaining to a single point in time. Therefore, the lack of additional evaluation of cortisol in 24-hour urine could be considered as a limitation of our study. A further limitation is that insulin resistance/HOMA-IR was only available in PCOS patients.

In conclusion, this prospective case-control study revealed the following main findings: there were no differences in laboratory stress biomarkers between PCOS women and controls. However, PCOS women suffered from higher perceived stress and a lower quality of life. A higher BMI and hirsutism seemed to influence perceived stress mainly in obese PCOS women. Last not least, perceived stress was positively correlated to prolactin levels in PCOS patients.

## Data availability statement

The raw data supporting the conclusions of this article will be made available by the authors, without undue reservation.

## Ethics statement

The studies involving human participants were reviewed and approved by Ethics Committee of the Medical University of Vienna, Vienna, Austria. The patients/participants provided their written informed consent to participate in this study.

## Author contributions

M-LM and JO contributed to conception and design of the study and data acquisition. RM and CS contributed to the analysis and the interpretation of data. JO performed the statistical analysis. MM wrote the first draft of the manuscript. All authors wrote sections of the manuscript. All authors contributed to manuscript revision, read, and approved the submitted version.

## References

[1] Cinar N Kizilarslanoglu MC Harmanci A Aksoy DY Bozdag G Demir B. Depression, anxiety and cardiometabolic risk in polycystic ovary syndrome. Hum Reprod (2011) 26(12):3339–45. doi: 10.1093/humrep/der338 21984577

[2] Scaruffi E Gambineri A Cattaneo S Turra J Vettor R Mioni R. Personality and psychiatric disorders in women affected by polycystic ovary syndrome. Front Endocrinol (Lausanne) (2014) 5:185. doi: 10.3389/fendo.2014.00185 25429283 PMC4228916

[3] Wang Y Ni Z Li K. The prevalence of anxiety and depression of different severity in women with polycystic ovary syndrome: a meta-analysis. Gynecol Endocrinol (2021) 37(12):1072–8. doi: 10.1080/09513590.2021.1942452 34165386

[4] Benson S Arck PC Tan S Hahn S Mann K Rifaie N. Disturbed stress responses in women with polycystic ovary syndrome. Psychoneuroendocrinology (2009) 34(5):727–35. doi: 10.1016/j.psyneuen.2008.12.001 19150179

[5] Veltman-Verhulst SM Boivin J Eijkemans MJ Fauser BJ. Emotional distress is a common risk in women with polycystic ovary syndrome: a systematic review and meta-analysis of 28 studies. Hum Reprod Update (2012) 18(6):638–51. doi: 10.1093/humupd/dms029 22824735

[6] Benjamin JJ Kuppusamy M Koshy T Kalburgi Narayana M Ramaswamy P. Cortisol and polycystic ovarian syndrome - a systematic search and meta-analysis of case-control studies. Gynecol Endocrinol (2021) 37(11):961–7. doi: 10.1080/09513590.2021.1908254 33818258

[7] Nouri K Litschauer B Huber JC Buerkle B Tiringer D Tempfer CB. Saliva cortisol levels and subjective stress are not associated with number of oocytes after controlled ovarian hyperstimulation in patients undergoing in vitro fertilization. Fertil steril (2011) 96(1):69–72. doi: 10.1016/j.fertnstert.2011.04.063 21620394

[8] Vining RF, McGinley RA, maksvytis JJ, ho KY. salivary cortisol: a better measure of adrenal cortical function than serum cortisol. Ann Clin Biochem (1983) 20(Pt 6):329–35. doi: 10.1177/000456328302000601 6316831

[9] Tsubouchi H Nakai Y Toda M Morimoto K Chang YS Ushioda N. Change of salivary stress marker concentrations during pregnancy: maternal depressive status suppress changes of those levels. J obstet gynaecol Res (2011) 37(8):1004–9. doi: 10.1111/j.1447-0756.2010.01473.x 21463431

[10] Hong RH Yang YJ Kim SY Lee WY Hong YP. Determination of appropriate sampling time for job stress assessment: the salivary chromogranin a and cortisol in adult females. J Prev Med Public Health = Yebang Uihakhoe chi (2009) 42(4):231–6. doi: 10.3961/jpmph.2009.42.4.231 19675399

[11] Wagner J Cik M Marth E Santner BI Gallasch E Lackner A. Feasibility of testing three salivary stress biomarkers in relation to naturalistic traffic noise exposure. Int J hygiene Environ Health (2010) 213(2):153–5. doi: 10.1016/j.ijheh.2009.08.004 19758843

[12] Ohashi J Katsura T. The effects of coaching on salivary cortisol stress marker in mothers with young children, a randomized controlled trial. J Rural Med JRM / Japanese Assoc Rural Med (2015) 10(1):20–8. doi: 10.2185/jrm.2891r PMC4571746 26380587

[13] University Monash. International evidence-based guideline for the assessment and management of polycystic ovary syndrome(2018). Available at: https://www.monash.edu/:data/assets/pdf_file/0004/1412644/PCOS_Evidence-Based-Guidelines_20181009.pdf (Accessed April 4th 2023).

[14] McDaid D Park AL Wahlbeck K. The economic case for the prevention of mental illness. Annu Rev Public Health (2019) 40:373–89. doi: 10.1146/annurev-publhealth-040617-013629 30601725

[15] Thapar A Eyre O Patel V Brent D. Depression in young people. Lancet (2022) 400(10352):617–31. doi: 10.1016/S0140-6736(22)01012-1 35940184

[16] Alvarez GE Beske SD Ballard TP Davy KP. Sympathetic neural activation in visceral obesity. Circulation (2002) 106(20):2533–6. doi: 10.1161/01.cir.0000041244.79165.25 12427647

[17] Lansdown A Rees DA. The sympathetic nervous system in polycystic ovary syndrome: a novel therapeutic target? Clin Endocrinol (2012) 77(6):791–801. doi: 10.1111/cen.12003 22882204

[18] Stepto NK Cassar S Joham AE Hutchison SK Harrison CL Goldstein RF. Women with polycystic ovary syndrome have intrinsic insulin resistance on euglycaemic-hyperinsulaemic clamp. Hum Reprod (2013) 28(3):777–84. doi: 10.1093/humrep/des463 23315061

[19] Alvarez-Blasco F Botella-Carretero JI San Millan JL Escobar-Morreale HF. Prevalence and characteristics of the polycystic ovary syndrome in overweight and obese women. Arch Intern Med (2006) 166(19):2081–6. doi: 10.1001/archinte.166.19.2081 17060537

[20] Kakoly NS Earnest A Teede HJ Moran LJ Joham AE. The impact of obesity on the incidence of type 2 diabetes among women with polycystic ovary syndrome. Diabetes Care (2019) 42(4):560–7. doi: 10.2337/dc18-1738 30705063

[21] Dokras A Stener-Victorin E Yildiz BO Li R Ottey S Shah D. Androgen excess- polycystic ovary syndrome society: position statement on depression, anxiety, quality of life, and eating disorders in polycystic ovary syndrome. Fertil steril (2018) 109(5):888–99. doi: 10.1016/j.fertnstert.2018.01.038 29778388

[22] Cohen S Kamarck T Mermelstein R. A global measure of perceived stress. J Health Soc Behav (1983) 24(4):385–96. doi: 10.2307/2136404 6668417

[23] Ware JE Jr. Gandek B Kosinski M Aaronson NK Apolone G Brazier J. The equivalence of sf-36 summary health scores estimated using standard and country-specific algorithms in 10 countries: results from the iqola project. international quality of life assessment. J Clin Epidemiol (1998) 51(11):1167–70. doi: 10.1016/S0895-4356(98)00108-5 9817134

[24] Rotterdam ESHRE/ASRM-Sponsored PCOS consensus workshop group. Revised 2003 consensus on diagnostic criteria and long-term health risks related to polycystic ovary syndrome (Pcos). Hum Reprod (2004) 19(1):41–7. doi: 10.1093/humrep/deh098 14688154

[25] Matthews DR Hosker JP Rudenski AS Naylor BA Treacher DF Turner RC. Homeostasis model assessment: insulin resistance and beta-cell function from fasting plasma glucose and insulin concentrations in man. Diabetologia (1985) 28(7):412–9. doi: 10.1007/BF00280883 3899825

[26] Reis D Lehr D Heber E Ebert DD. The German version of the perceived stress scale (Pss-10): evaluation of dimensionality, validity, and measurement invariance with exploratory and confirmatory bifactor modeling. Assessment (2019) 26(7):1246–59. doi: 10.1177/1073191117715731 28627220

[27] Klein EM Brahler E Dreier M Reinecke L Muller KW Schmutzer G. The German version of the perceived stress scale - psychometric characteristics in a representative German community sample. BMC Psychiatry (2016) 16:159. doi: 10.1186/s12888-016-0875-9 27216151 PMC4877813

[28] Ware JE Jr. Sherbourne CD. The mos 36-item short-form health survey (Sf-36). i. conceptual framework and item selection. Med Care (1992) 30(6):473–83. 1593914

[29] Ferriman D Gallwey JD. Clinical assessment of body hair growth in women. J Clin Endocrinol Metab (1961) 21:1440–7. doi: 10.1210/jcem-21-11-1440 13892577

[30] Doshi A Zaheer A Stiller MJ. A comparison of current acne grading systems and proposal of a novel system. Int J Dermatol (1997) 36(6):416–8. doi: 10.1046/j.1365-4362.1997.00099.x 9248884

[31] Hager M Ott J Marschalek J Marschalek ML Kinsky C Marculescu R. Basal and dynamic relationships between serum anti-mullerian hormone and gonadotropins in patients with functional hypothalamic amenorrhea, with or without polycystic ovarian morphology. Reprod Biol Endocrinol (2022) 20(1):98. doi: 10.1186/s12958-022-00961-y 35787707 PMC9251918

[32] Chojnowska S Ptaszynska-Sarosiek I Kepka A Knas M Waszkiewicz N. Salivary biomarkers of stress, anxiety and depression. J Clin Med (2021) 10(3):517. doi: 10.3390/jcm10030517 33535653 PMC7867141

[33] Giacomello G Scholten A Parr MK. Current methods for stress marker detection in saliva. J Pharm BioMed Anal (2020) 191:113604. doi: 10.1016/j.jpba.2020.113604 32957066 PMC7474833

[34] Basu BR Chowdhury O Saha SK. Possible link between stress-related factors and altered body composition in women with polycystic ovarian syndrome. J Hum Reprod Sci (2018) 11(1):10–8. doi: 10.4103/jhrs.JHRS_78_17 PMC5892097 29681710

[35] Mezzullo M Fanelli F Di Dalmazi G Fazzini A Ibarra-Gasparini D Mastroroberto M. Salivary cortisol and cortisone responses to short-term psychological stress challenge in late adolescent and young women with different hyperandrogenic states. Psychoneuroendocrinology (2018) 91:31–40. doi: 10.1016/j.psyneuen.2018.02.022 29522931

[36] Tock L Carneiro G Pereira AZ Tufik S Zanella MT. Adrenocortical production is associated with higher levels of luteinizing hormone in nonobese women with polycystic ovary syndrome. Int J Endocrinol (2014) 2014:620605. doi: 10.1155/2014/620605 24895496 PMC4033536

[37] Luboshitzky R Ishai A Shen-Or Z Herer P. Evaluation of the pituitary-adrenal axis in hyperandrogenic women with polycystic ovary syndrome. Neuro Endocrinol Lett (2003) 24(3-4):249–54. 14523365

[38] Martikainen H Salmela P Nuojua-Huttunen S Perala J Leinonen S Knip M. Adrenal steroidogenesis is related to insulin in hyperandrogenic women. Fertil steril (1996) 66(4):564–70. doi: 10.1016/s0015-0282(16)58568-9 8816617

[39] Kamel N Tonyukuk V Emral R Corapcioglu D Bastemir M Gullu S. Role of ovary and adrenal glands in hyperandrogenemia in patients with polycystic ovary syndrome. Exp Clin Endocrinol Diabetes (2005) 113(2):115–21. doi: 10.1055/s-2004-830540 15772904

[40] Lanzone A Petraglia F Fulghesu AM Ciampelli M Caruso A Mancuso S. Corticotropin-releasing hormone induces an exaggerated response of adrenocorticotropic hormone and cortisol in polycystic ovary syndrome. Fertil steril (1995) 63(6):1195–9. doi: 10.1016/s0015-0282(16)57596-7 7750588

[41] Morin-Papunen LC Vauhkonen I Koivunen RM Ruokonen A Tapanainen JS. Insulin sensitivity, insulin secretion, and metabolic and hormonal parameters in healthy women and women with polycystic ovarian syndrome. Hum Reprod (2000) 15(6):1266–74. doi: 10.1093/humrep/15.6.1266 10831553

[42] Pasquali R Gambineri A. Cortisol and the polycystic ovary syndrome. Expert Rev Endocrinol Metab (2012) 7(5):555–66. doi: 10.1586/eem.12.42 30780898

[43] Khafagy G El Sayed I Abbas S Soliman S. Perceived stress scale among adolescents with polycystic ovary syndrome. Int J Womens Health (2020) 12:1253–8. doi: 10.2147/IJWH.S279245 PMC7778675 33402850

[44] Tabassum F Jyoti C Sinha HH Dhar K Akhtar MS. Impact of polycystic ovary syndrome on quality of life of women in correlation to age, basal metabolic index, education and marriage. PloS One (2021) 16(3):e0247486. doi: 10.1371/journal.pone.0247486 33690645 PMC7946178

[45] Coffey S Bano G Mason HD. Health-related quality of life in women with polycystic ovary syndrome: a comparison with the general population using the polycystic ovary syndrome questionnaire (Pcosq) and the short form-36 (Sf-36). Gynecol Endocrinol (2006) 22(2):80–6. doi: 10.1080/09513590600604541 16603432

[46] Kumarapeli V Seneviratne Rde A Wijeyaratne C. Health-related quality of life and psychological distress in polycystic ovary syndrome: a hidden facet in south Asian women. BJOG (2011) 118(3):319–28. doi: 10.1111/j.1471-0528.2010.02799.x 21134104

[47] Brady C Mousa SS Mousa SA. Polycystic ovary syndrome and its impact on women's quality of life: more than just an endocrine disorder. Drug Healthc Patient Saf (2009) 1:9–15. doi: 10.2147/dhps.s4388 21701605 PMC3108690

[48] Aversa A La Vignera S Rago R Gambineri A Nappi RE Calogero AE. Fundamental concepts and novel aspects of polycystic ovarian syndrome: expert consensus resolutions. Front Endocrinol (Lausanne) (2020) 11:516. doi: 10.3389/fendo.2020.00516 32849300 PMC7431619

[49] Yin X Ji Y Chan CLW Chan CHY. The mental health of women with polycystic ovary syndrome: a systematic review and meta-analysis. Arch Womens Ment Health (2021) 24(1):11–27. doi: 10.1007/s00737-020-01043-x 32514730

[50] Karsten MDA Wekker V Groen H Painter RC Mol BWJ Laan ETM. The role of pcos in mental health and sexual function in women with obesity and a history of infertility. Hum Reprod Open (2021) 2021(4):hoab038. doi: 10.1093/hropen/hoab038 34877412 PMC8643501

[51] Scaruffi E Franzoi IG Civilotti C Guglielmucci F La Marca L Tomelini M. Body image, personality profiles and alexithymia in patients with polycystic ovary syndrome (Pcos). J Psychosom Obstet Gynaecol (2019) 40(4):294–303. doi: 10.1080/0167482X.2018.1530210 30398405

[52] Himelein MJ Thatcher SS. Depression and body image among women with polycystic ovary syndrome. J Health Psychol (2006) 11(4):613–25. doi: 10.1177/1359105306065021 16769740

[53] Keegan A Liao LM Boyle M. 'Hirsutism': a psychological analysis. J Health Psychol (2003) 8(3):327–45. doi: 10.1177/13591053030083004 14670212

[54] Brower MA Hai Y Jones MR Guo X Chen YI Rotter JI. Bidirectional mendelian randomization to explore the causal relationships between body mass index and polycystic ovary syndrome. Hum Reprod (2019) 34(1):127–36. doi: 10.1093/humrep/dey343 PMC6295958 30496407

[55] Jiang X Deng Q Stener-Victorin E. Is there a shared genetic basis and causal relationship between polycystic ovary syndrome and psychiatric disorders: evidence from a comprehensive genetic analysis. Hum Reprod (2021) 36(8):2382–91. doi: 10.1093/humrep/deab119 34051085

[56] Noushad S Ahmed S Ansari B Mustafa UH Saleem Y Hazrat H. Physiological biomarkers of chronic stress: a systematic review. Int J Health Sci (Qassim) (2021) 15(5):46–59. 34548863 PMC8434839

[57] Belda X Fuentes S Daviu N Nadal R Armario A. Stress-induced sensitization: the hypothalamic-Pituitary-Adrenal axis and beyond. Stress (2015) 18(3):269–79. doi: 10.3109/10253890.2015.1067678 26300109

