# Supplementary material for: A case-control study about markers of stress in normal-/overweight women with polycystic ovary syndrome and in controls
Source: Front Endocrinol (Lausanne). 2023 May 16;14:1173422. doi: 10.3389/fendo.2023.1173422 (PMC10231031; doi:10.3389/fendo.2023.1173422)
Supplement: Supplementary file 1 [file Table_1.docx]

**Table 5**. Basic patient characteristics and outcome parameters in lean/normal weight and overweight/obese PCOS patients.

|  | **Lean/normal weight PCOS patients** | **Overweight/obese PCOS patients** | **p** |
| --- | --- | --- | --- |
| Age (years) | 25 (20;31) | 25 (22;30) | 0.989 |
| BMI (kg/m^2^) | 22.0 (18.6;23.0) | 34.8 (32.5;40.4) | <0.001 |
| Ferriman Gallwey Score | 8 (0;14) | 13 (8;18) | 0.188 |
| GAGS | 0 (0;11) | 7 (3;10) | 0.309 |
| LH (mlU/mL) | 14.2 (9.8;17.3) | 11.8 (8.2;13.3) | 0.301 |
| FSH (mlU/mL) | 5.6 (4.7;7.7) | 6.2 (4.9;7.5) | 0.723 |
| LH: FSH ratio | 2.2 (1.6;3.1) | 2.1 (1.1;2.7) | 0.415 |
| Testosterone (ng/mL) | 0.45 (0.36;0.58) | 0.60 (0.49;0.76) | 0.209 |
| SHBG (nmol/L) | 62.3 (33.1;99.0) | 31.0 (20.2;36.0) | 0.022 |
| Free androgen index | 0.76 (0.59;1.15) | 2.00 (1.56;3.04) | 0.007 |
| DHEA-S (µg/ml) | 2.84 (2.14;3.08) | 3.32 (3.01;4.65) | 0.169 |
| Prolactin (ng/mL) | 14.1 (8.9;21.7) | 12.6 (8.9; 17.0) | 0.820 |
| AMH (ng/mL) | 10.40 (5.96;13.61) | 7.23 (4.33-8.30) | 0.022 |

Data are provided as median (interquartile range) for numerical parameters and as number (frequency) for categorical parameters

Abbreviations used: BMI, body mass index; HOMA-IR, HOMA index of insulin resistance; GAGS, global acne grading system; LH, luteinizing hormone; FSH, follicle stimulating hormone; DHEA-S, dehydroepiandrosterone-sulfate; SHBG, sexual hormone binding globulin; AMH, anti-Mullerian hormone
